# The National Hospital for the Paralysed

**Published:** 1902-04-05

**Authors:** 


### The Institutional Workshop.

### THE NATIONAL HOSPITAL FOR THE PARALYSED.

ANNUAL MEETING.

The meeting at the National Hospital for the Paralysed and Epileptic on Wednesday, March 26th, was, as the Chairman remarked, one of the shortest held in recent times at this hospital. The business occupied precisely 35 minutes. Earl Dudley took the chair, and with him at the table were the following members of the newly-elected board: The Rev. Dr. Wace, Sir James Paget, Sir Felix Semon, Dr. Ormerod, and Mr. F. O. Macmillan. The attendance of governors was poor, only about ten being present; two of these were ladies. The tone of the meeting was quiet and dignified, and the only voices from the body of the room were those of Mr. Nelson and Mr. Burford Rawlings.

The Chairman opened the proceedings by a courteous refusal to be in any way responsible for the report and accounts before the meeting.

The Chairman's Speech.

Earl Dudley said: Although this is an occasion on which the affairs of the hospital are usually entered upon fully, it will be impossible to do so as in previous years, the reason being that the present members of the Board have only been in office a very few weeks. The report which has been laid before the governors is a report of the proceedings of administration of the late board, and it is therefore impossible for the board to-day to assume responsibility or to attempt to discuss it as they would if it had been one of their own producing. The same thing applies to the accounts. I think that is a reasonable position to take up, and I am sure every governor will understand our position. I think I may perhaps be allowed to refer to the work which for the last six weeks or two months has been done by Mr. Danvers Power, who is unfortunately laid up and cannot be here to-day. It is quite true to say that the hospital has been exceedingly fortunate in finding a gentleman who in a very exceptional time has been willing to devote his whole time to the work. Unless we had been fortunate enough to secure his services during the past few weeks the difficulties would have been greater than they have been. I feel sure that every governor will feel gratitude for what he has done. We have secured the services, as secretary, of Mr. Kirby, and we have every reason to hope that he will prove of valuable assistance to this hospital. Now the first thing upon our agenda is naturally the consideration of the previous minutes. It is usual on this occasion that the minutes of the previous meeting should be read. Well, it will be within the recollection of all that we have had three important meetings, and the minutes occupy, I am told, three closely-written pages of the minute-book. I do not suppose anybody wants to listen to the reading of three closely-written pages, setting forth what has happened in the past few weeks. I therefore suggest that we take them as read.

Mr. Nelson said that it seemed hardly necessary to read minutes which were familiar to a large number of governors.

The Chairman: I will guarantee that they are in order, and with your permission I will sign them as if they had been read.

Dr. Wace moved, without comment, that the annual report for 1891 be received, and Sir Felix Semon formally seconded the motion.

One Word.

Mr. Nelson said he would like to say one word. The report, he understood, was inherited from the last Board, but it seemed to him that it would be better if longer notice were given of any important business. It had come to his knowledge absolutely accidentally that a certain number of gentlemen had been nominated for the Board. Might he venture to suggest that the Board should take into consideration the question of letting the report be in the hands of the governors some time before the meeting? He had never seen this report before. There might be grave errors in it, but he could not tell, having just picked it up from a chair. Several members of the Board assured Mr. Nelson that this would be done, and after a further criticism, this time on the rules governing the nominations for the Board, from Mr. Nelson, the Chairman put the motion, that the report he received, and, Mr. Nelson having seconded, it was carried.

The President.

The election of the President was the next business, and on the motion of Dr. Wace, seconded by Sir Felix Semon, Earl Dudley was unanimously elected. He accepted the office, and said he hoped the governors would have no reason to repent of their choice; he was prepared to do all in his power for the good of the hospital.

The Auditors.

The next business gave rise to a somewhat painful discussion. Dr. Wace said there was a paragraph in Sir Edward Fry's Committee's report which amounted to severe censure of the auditors, and the Board proposed that Messrs. Price and Waterhouse be elected in their place. Their names were known to every business man.

A discussion followed in which Mr. Nelson and Mr. Burford Rawlings took part. The former said he was not aware of any censure, though he would take Dr. Wace's word for it. Dr. Wace read the paragraph, which stated that the late auditors never drew attention to the rules governing the accounts, and added that there had been irregularities. At the word irregularities Mr. Burford Rawlings rose, and said he was aware of no irregularities. On the contrary, he thought it would be found that there was a paragraph stating that the finances were administered wisely and well. The Chairman said he rather wished Mr. Burford Rawlings had selected another opportunity for making that statement; the business of that meeting would be better administered if they limited themselves to the question in hand. The words in Sir Edward Fry's report had been read. It surely need not be discussed whether they amounted to a censure or not. He supposed it was for the Board to suggest to the meeting the appointment of auditors whom they considered fitting for the post. That being so, and the gentlemen in question being in every way suitable, there was no necessity to discuss whether the previous auditors were guilty or not. They had the power to change without going into reasons. By going into arguments on matters everyone wanted to bury, only harm could be done. There was no reason for doubting that the proposed appointment was a good one, and he hoped it would be made without raising questions about the fitness of late officers. Dr. Wace said it was quite right to say that the funds had been properly dealt with. There was no charge whatever against the late Board with respect to them, but there were certain points the auditors ought to have attended to, and did not. Mr. Burford Rawlings was quite right in saying that the report paid a high tribute to the administration of the funds.

Mr. Burford Rawlings: Thank you very much. I only thought it might convey a wrong impression.

The Bye-laws.

The next point on the agenda was "to authorise the continuance of the existing bye-laws so far as practicable under the new rules." The Chairman said the Board was revising the bye-laws, but they had not had time to finish, and were not yet in a position to lay their proposals before the governors. They therefore suggested that, so far as possible, the old ones should remain in force. When he said so far as possible, he meant that the old bye-laws were based upon the office of secretary-director. That office did not now exist, and it was obvious that the bye-laws needed modification. The Board hoped the governors would give them the justification to utilise the old ones for the present as far as possible and await their final consideration in their new form, which it was hoped to lay before them. Dr. Wace formally moved, and Mr. Royle seconded, the continuance, as far as possible, of the old bye-laws.

At this point, the only thing left on the agenda paper being the transaction of any other business admissible by the rules, the Chairman said that personally he knew of no other business, but if the meeting had any point to raise or question to ask, the Board would be glad to consider it.

Two Questions.

Mr. Nelson asked whether consideration had been given to the question of receiving money from patients who might be in a position to contribute something in return for the benefits they had received. He knew there were two opinions and that it was a vexed question; he hoped the Board would give their close attention to it. He had a great deal of sympathy for the very large class who were a little too well off to go to the hospital, but by no means rich enough to get medical advice at a high cost. It was a very expensive luxury. Mr. Nelson's next question referred to Mr. Burford Rawlings, and he prefaced it by saying that he knew very little of that gentleman, and was in no way prompted by him. He wanted to know to what extent, if any, Mr. Rawlings was under an obligation to the hospital, and whether he was in any way bound, supposing he saw his way to taking other employment.

Dr. Wace replied. He said the governors had done what they considered to be justice, and Mr. Rawlings was perfectly free to do what he liked. As to paying patients, it was a difficult question of policy on which varying opinions were held. At present patients were received into the wards at a guinea a week, and whether anything else might be done in that way he did not know. It was for the board to consider.

A vote of thanks to the Chairman was proposed by Mr. Nelson, and seconded by Sir Felix Semon, and the meeting broke up into little groups for private discussion.

